# Supplementary material for: Pharmacist-led rapid uptitration clinic in heart failure patients with reduced ejection fraction: Our experience within a virtual ward
Source: PLOS Digit Health. 2025 Jun 5;4(6):e0000868. doi: 10.1371/journal.pdig.0000868 (PMC12140189; doi:10.1371/journal.pdig.0000868)
Supplement: Attachment — Submitted filename: Comments for Dr H. Alhakem_PDIG-D-24-00601 (1).pdf [file pdig.0000868.s003.pdf]

Comments to the author

The manuscript titled "*Evaluation of a pharmacist-led rapid uptitration clinic for guideline-directed medical therapy in heart failure patients with reduced ejection fraction within a virtual ward*" presents a retrospective study employing a single-group pre-post design to evaluate the effectiveness of a prescribing pharmacist-led clinic in the rapid uptitration of Guidelines Directed Medical Therapy (GDMT) in patients with heart failure with reduced ejection fraction (HFrEF) within a virtual ward setting. The study assessed clinical outcomes of 86 patients at baseline, following discharge from the virtual ward (typically after 4 weeks), and at 3-6 months post-discharge. Improvements were seen in terms of NYHA scores, cardiac systolic function, optimal Medical Therapy (OMT) scores. The information provided in this work could be of scientific value.

However, some methodological, reporting, formal and substantial aspects are discussed below.

General comment

This study could be of interest for PLOS ONE Digital, as it presents a very useful and innovative approach (virtual wards) in transforming heart failure management by combining rapid medication optimisation with remote monitoring to improve patient outcomes.

However, although the study outlines an important way to improve adherence to drug therapies from the comfort of home, reduce hospitalisations and improve the quality of life of heart failure patients, the study presents issues that jeopardize its scientific validity, such as the lack of a standardized format and the lack of completeness in presenting findings.

TITLE

Title reflects what you did, but reconsider the proper use of the term "evaluation".

ABSTRACT

The abstract is readable, and it provides an acceptable overview of your experiment. I would suggest to avoid the last sentence: "Although our findings reached statistical significance, further investigation by adjusting the intervention timeline to allow for stabilisation of LVEF may offer additional benefits."

This statement is not useful to your readers, do not limit your study to a problem of statistical significance. Singular fixation on $p<0.05$ (or lower) can be misleading, especially in complex clinical setting where large sample size are usually difficult to achieve.

INTRODUCTION

In general, it is understandable, but it is asymmetrical in its main parts and does not introduce the topic extensively. Moreover, I would suggest moving Table 1 elsewhere in the paper.

METHODOLOGY

Overall, this section is clear but could be presented better along its main parts. In depth, the level of detail provided in the statistical analysis plan is not enough. For example, how did you present categorical/numerical variables? What about the level of significance and the confidence interval? Have you used a test of normality to evaluate the distribution of your data?

Lastly, guide the reader towards a deeper understanding of the supplementary material, reinforcing your commitment to transparency in data sharing.

RESULTS

This section is readable.

Please, do not undervalue the importance of presenting the p-value in the right way. This has always to be presented with a measure of effect estimation and/or with a confidence interval, to contextualize the plausibility and the precision of the effect.

DISCUSSION AND CONCLUSION

Discussion is readable, but does not argue enough the burden evidence available so far around this topic. The findings of your experiment could have not only clinical but also general public health implications.
