# Supplementary material for: Pharmacist-led rapid uptitration clinic in heart failure patients with reduced ejection fraction: Our experience within a virtual ward
Source: PLOS Digit Health. 2025 Jun 5;4(6):e0000868. doi: 10.1371/journal.pdig.0000868 (PMC12140189; doi:10.1371/journal.pdig.0000868)
Supplement: Attachment — Submitted filename: Response to Reviewers.docx [file pdig.0000868.s005.docx]

**Title**

We have revised the title from "Evaluation of a pharmacist-led rapid uptitration clinic for guideline-directed medical therapy in heart failure patients with reduced ejection fraction within a virtual ward" to "Pharmacist-led rapid uptitration clinic in heart failure patients with reduced ejection fraction: our experience within a virtual ward" to better reflect the study scope.

**Abstract**

We have removed the sentence: “Although our findings reached statistical significance, further investigation by adjusting the intervention timeline to allow for stabilisation of LVEF may offer additional benefits.”

**Introduction**

We have restructured the introduction to enhance its flow and clarity by:

1. Providing background to contextualise the study.

2. Defining the problem and its significance.

3. Including a brief review of key literature.

4. Concluding with the project’s aim and its impact on addressing gaps in care.

Table 1 has been moved to the Methods section as suggested.

**Methodology**

We have expanded the statistical analysis section with the following additions:

• Used the Kolmogorov-Smirnov (K-S) test to assess normality.

• Used a paired t-test due to the non-normal distribution of LVEF% differences post-intervention.

• Included statistical equations and justification for selecting the paired t-test.

• Provided explicit reference to Supplementary S2 Appendix for full statistical details.

• Added a statement directing readers to the raw dataset in Supplementary S1 Appendix.

**Results**

We have revised this to include odds ratios, corresponding 95% confidence intervals, and p-values for the key outcomes: prediction of improvement in LVEF >35%, Optimal Medical Therapy (OMT) score of 8, and mortality at 90 days post-uptitration. This addition provides better context for the plausibility and precision of the reported effects, in line with your recommendation.

**Discussion and Conclusion**

We have expanded the discussion by:

• Referring to major studies (CHAMP-HF, BIOSTAT-CHF) that highlight the benefits of aggressive GDMT optimisation and demonstrating the survival benefit of early GDMT initiation.

• Incorporating evidence from the TIM-HF2 trial supporting the role of remote monitoring in reducing mortality and hospitalisations.

• Highlighting the prevalence of comorbidities (T2DM, CKD) in our cohort and linking findings to the DAPA-HF and EMPEROR-Reduced trials, emphasising the broader public health impact of comprehensive GDMT initiation.
